# Biological Evaluation, Phytochemical Screening, and Fabrication of *Indigofera linifolia* Leaves Extract-Loaded Nanoparticles

**DOI:** 10.3390/molecules27154707

**Published:** 2022-07-23

**Authors:** Muhammad Talha, Noor Ul Islam, Muhammad Zahoor, Abdul Sadiq, Asif Nawaz, Farhat Ali Khan, Naila Gulfam, Saleh A. Alshamrani, Mohammed H. Nahari, Mohammed Abdulrahman Alshahrani, Mater H. Mahnashi, Syed Shams ul Hassan

**Affiliations:** 1 Department of Biochemistry, University of Malakand, Chakdara 18800, Pakistan; livingontheedge36837@gmail.com; 2 Department of Chemistry, University of Malakand, Chakdara 18800, Pakistan; nooruomchem@gmail.com; 3 Department of Pharmacy, University of Malakand, Chakdara 18800, Pakistan; sadiquom@yahoo.com (A.S.); asifnawaz2445@gmai.com (A.N.); 4 Department of Pharmacy, Shaheed Benazir Bhuto University, Sheringal 18000, Pakistan; farhatkhan2k9@yahoo.com; 5 Department of Zoology, Jinnah College for Women, University of Peshawar, Peshawar 182300, Pakistan; nailazoo@yahoo.com; 6 Department of Clinical Laboratory Sciences, College of Applied Medical Sciences, Najran University, Najran 61441, Saudi Arabia; 7 Department of Clinical Laboratory Sciences, Najran University, Najran 61441, Saudi Arabia; mhnahari@nu.edu.sa; 8 Department of Clinical Laboratory Sciences, Faculty of Applied Medical Sciences, Najran University, P.O. Box 1988, Najran 61441, Saudi Arabia; maalshahrani@nu.edu.sa; 9 Department of Pharmaceutical Chemistry, College of Pharmacy, Najran University, Najran 61441, Saudi Arabia; matermaha@gmail.com; 10 Shanghai Key Laboratory for Molecular Engineering of Chiral Drugs, School of Pharmacy, Shanghai Jiao Tong University, Shanghai 200240, China; shams1327@yahoo.com; 11 Department of Natural Product Chemistry, School of Pharmacy, Shanghai Jiao Tong University, Shanghai 200240, China

**Keywords:** *Indigofera linifolia*, antioxidant, antidiabetic, nanoparticles

## Abstract

*Indigofera linifolia* is a medicinally important plant, and by virtue of its rich phytochemical composition, this plant is widely used as essential component in traditional medication systems. Due to its wide range of medicinal applications, the extract-loaded chitosan (Ext+Ch), extract-loaded PEG (Ext+PEG), and extract-loaded locust bean gum (Ext+LGB) nanoparticles (NPs) were prepared in the present study. The prepared NPs were then evaluated for their antibacterial, antioxidant, and antidiabetic potentials. Antibacterial activities of the crude extract and the synthesized NPs were performed following standard procedures reported in the literature. The antioxidant capabilities of extract and NPs were evaluated using DPPH free radical scavenging assay. The antidiabetic potential of the samples was evaluated against α-amylase and α-glucosidase. Ext+PEG NPs showed more potent antibacterial activity against the selected strains of bacteria with the highest activity against *Escherichia coli*. The lowest antibacterial potential was observed for Ext+LGB NPs. The Ext+LGB NPs IC_50_ value of 39 μg/mL was found to be the most potent inhibitor of DPPH free radicals. Ext+LGB NPs showed a greater extent of inhibition against α-glucosidase and α-amylase with an IC_50_ of 83 and 78 μg/mL, whereas for the standard acarbose the IC_50_ values recorded against the mentioned enzymes were 69 and 74 μg/mL, respectively. A high concentration of phenolics and flavonoids in the crude extract was confirmed through TPC and TFC tests, HPLC profiling, and GC–MS analysis. It was considered that the observed antibacterial, antidiabetic, and antioxidant potential might be due the presence of these phenolics and flavonoids detected. The plant could thus be considered as a potential candidate to be used as a remedy of the mentioned health complications. However, further research in this regard is needed to isolate the exact responsible compounds of the observed biological potentials exhibited by the crude extract. Further, toxicity and pharmacological evaluations in animal models are also needed to establish the safety or toxicity profile of the plant.

## 1. Introduction

From the beginning of human life on earth, humans have relied on medicinal plants to cure various diseases. However, in the 20th century, as the human civilization got more developed and along with the isolation of medicinally important compounds, they attempted to synthesize medicinally important compounds and their derivatives in laboratories. Although many active pharmaceutical ingredients have been prepared by the synthetic route, the importance of plants still cannot be ignored and that is reason that plants are constantly explored for novel compounds of biological importance [1]. Medicinal plants are the main source of pharmaceutical compounds and various valuable medicines that have been derived from them directly or indirectly [2,3]. As mentioned since ancient times, humans use plants, flowers, and insects to isolate different compounds, beneficial for health or curing diseases [4]. Natural compounds obtained from plants have been considered as the unchallenged foundations of new drug discoveries. However, the newest contest between combinatorial chemistry and computational drug design [5] has ended the supremacy of natural products in drug discovery. Many experts still believe that most natural products remain undiscovered and their utilization for pharmacological purposes still requires further investigations [6]. Even plants as reductants are constantly utilized by scientists to get the smallest size nanoparticles, which, due to their enhanced surface area offers better properties than their counterparts synthesized synthetically [7,8,9,10].

Nanoparticles (NPs) are stable colloidal particles [7], and owing to their larger surface area and small particle size they have unusual properties (physical and chemical) [8]. In pharmaceutical sciences, the solubility of certain drug ingredients in water leads to lower efficacies of the products. Using nanotechnology, the solubility, and consequently the bioavailability of such products could be enhanced, where for size reduction mostly plant extracts are used as reductant. Moreover, other related pharmacological properties such as a sustained delivery, a lower toxicity, and protection from physical and chemical degradation may also be enhanced through the use of nanotechnology [9,10]. Recently, the use of polymers in nanotechnology has drawn immense attention in many fields including the pharmaceutical industries. Natural polymers (proteins and polysaccharides) and synthetic polymers can be used in the preparation of NPs. NP fabrication through natural polymers do not need organic solvents and high shear forces. Due to potential biological activity, chitosan NPs have gained much interest in the medicinal field [7]. PEG (polyethylene glycol) is a polymer that is frequently used in drug administrations. It offers a wide range of therapeutic applications in cancer treatment. It is highly water-soluble and biocompatible, making it suitable in therapeutic drug administration. The US Food and Drug Administration has previously approved several PEG-conjugated medicines. Drug solubility, stability, and pharmacokinetic characteristics have been found improved by PEG conjugation [11]. Because of its wide application in food and medicines, locust bean gum (LBG) is one such biopolymer with enormous potential in medication formulations. Due to their benign nature, LBG-based formulations exhibit sustained release and can also be employed as muco-adhesives in some formulations. LBG microparticles and nanoparticles are now in the development stages to be used for pharmaceutical purposes. Several studies have looked into the various applications of LBG in drug delivery [12].

*Indigofera linifolia* herb is found throughout India and Pakistan where it is used for the treatment of various diseases. Its leaves are used as an antibacterial, an antioxidant, and are cytotoxic in lung cancer [13]. This plant has not been used in the fabrication of nanoparticles for medicinal uses.

In the current study, the GC–MS technique was used to assess phytochemical composition of *Indigofera linifolia* leaf extract. HPLC analysis were also performed to evaluate the phenolics and flavonoids in the extract. Additionally, the total phenolic content (TPC) and total flavonoid content (TFC) were also determined in the crude extract. The crude leaf extract was used in the fabrication of NPs using two natural polymers; chitosan and LGB, and one synthetic polymer, polyethylene glycol (PEG), to get extract loaded chitosan (Ext+Ch) NPs, extract-loaded PEG (Ext+PEG) NPs, and extract-loaded LGB (Ext+LGB) NPs. The crude extract and prepared NPs were evaluated for various biological potentials as well.

## 2. Results and Discussion

### 2.1. Preliminary Phytochemical Analysis

Plant-derived drugs have recently attracted a lot of attention due to their low side effects. Traditional and modern medicines (nutraceuticals, food supplements, folk remedies, and pharmaceutical intermediates) are totally or partially plant products [14]. Plants contain a number of secondary metabolites that are broadly classified into alkaloids, phenols, tannins, etc. A literature review has shown that plant leaves and roots are rich sources of secondary metabolites [15], and that is why they are used as such in a powdered form as medicines in traditional medication systems, whereas in modern medication systems, the isolated compounds or their derivatives are used.

To confirm the presence of broad phytochemical groups, a number of preliminary tests were used [14,15]. The preliminary phytochemical tests were positive, indicating that *Indigofera linifolia* is a rich source of phytochemicals (Table 1) and needs to be evaluated for different biological potentials.

### 2.2. TFC and TPC in the Crude Extract and Prepared NPs

**Figure 1:**
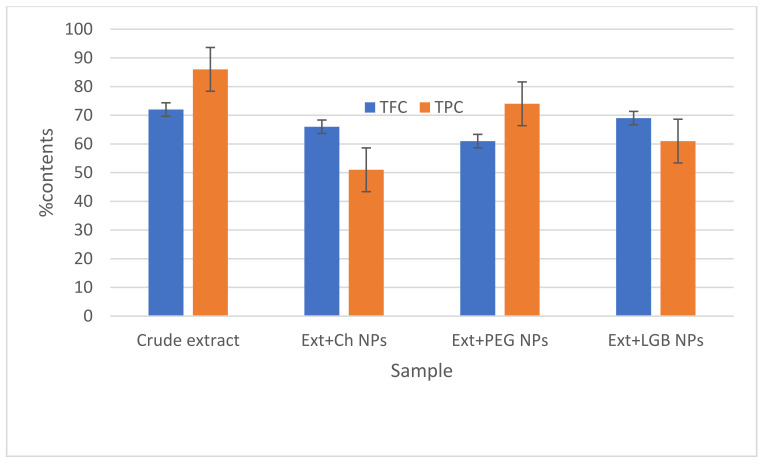
% TFC and TPC in *Indigofera linifolia* crude extract, Ext+Ch NPs, Ext+PEG NPs, and Ext+LGB NPs.

The TPC and TFC determined are graphically presented in Figure 1. The crude extract being a polymer-free product exhibited the highest TPC and TFC with values of 86% and 72%, respectively. After the addition of polymers, high TPCs were retained in Ext+PEG NPs (74%), whereas a high TFC was found in Ext+LGB NPs (69%).

### 2.3. Characterization of the Crude Extract

#### 2.3.1. HPLC-UV Analysis of the Crude Extract

**Figure 2:**
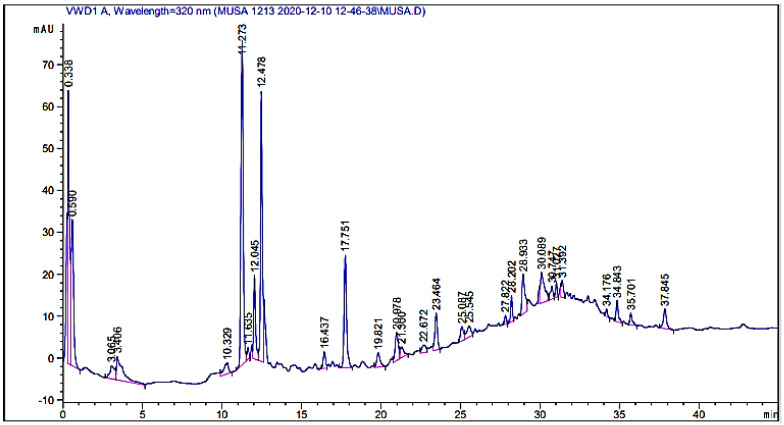
HPLC-UV chromatogram of the crude extract of *Indigofera linifolia* recorded at 320 nm.

In the herbal drug industry, the standardization and characterization of herbal products is a topic of immense scientific interest. With the introduction of sophisticated chromatographic techniques, there is a growing desire to create and develop simple, quick, convenient, and cost-effective standardization methods [16]. HPLC is a sensitive and precise tool that is frequently used for the qualitative assessment of plant extracts and their derived product/formulation [17]. A typical HPLC chromatogram of the crude extract is given in Figure 2, while the identified possible phytochemical compounds are given in Table 2. The names of the detected compounds along with the retention times are given as follows: mandelic acid (RT: 10.329), bis-HHDP-hex(pedunculagin) (RT: 11.273), caffeic acid (RT: 12.478), hydroxy benzoic acid (RT16.437), syringic acid (RT: 23.464), 5-o-dicaffeoylquinic acid (RT: 25.087), kaempferol-3-(caffeoyl-diglucoside)-7-rhamnosyl (RT: 25.545), kaempferol-3-(p-coumaroyl-diglucoside)-7-glucoside (RT: 27.822), chlorogenic acid (RT: 20.978), morin (RT: 22.672), Quercetin (RT: 30.089), Qurcetin-3-(caffeoyldiglucoside)-7-glucoside (RT: 31.027), quercitin-3-o-rutinoside (RT: 34.843), and glucose (RT: 37.845). The identification of these compounds is based on a comparison of their retention times with those of the available standards [18]. There are a number of peaks of unknown compounds as well that need further structural elucidations. The chromatograms show that the plant is a rich source of secondary metabolites.

#### 2.3.2. GC–MS Analysis

**Figure 3:**
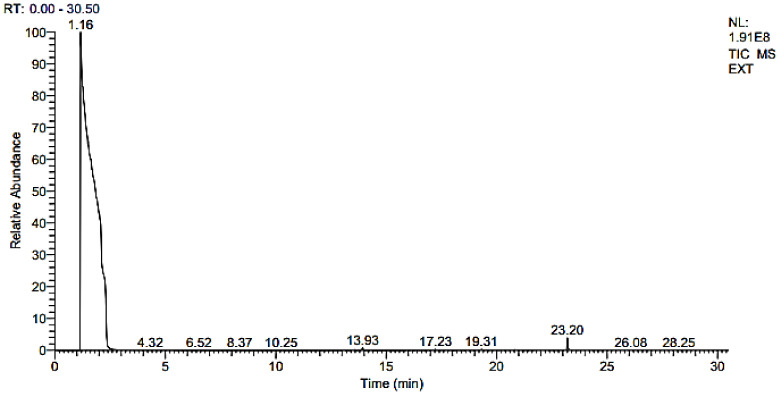
GC chromatogram of *Indigofera linifolia* leaves extract.

**Figure 4:**
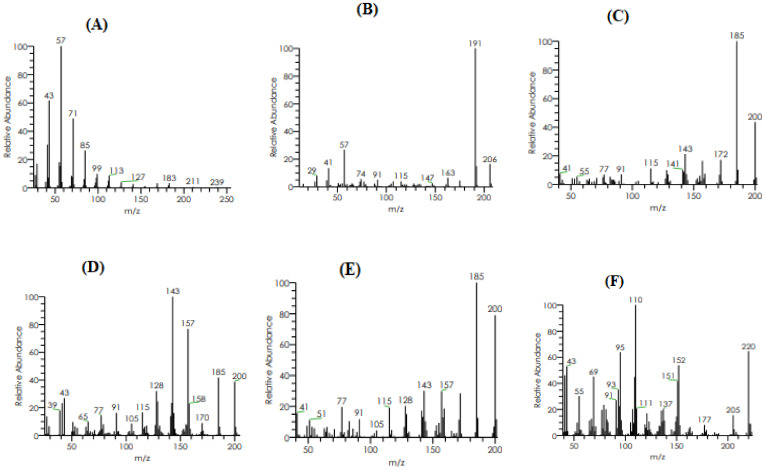

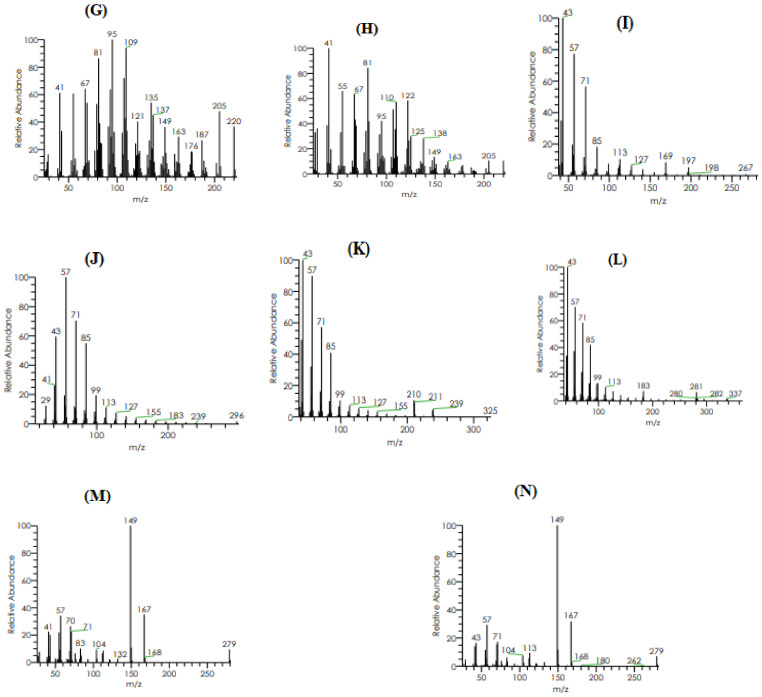
GC–MS chromatograms of detected compounds: (**A**) 2,6,10-Trimethylpentadecane, (**B**) Phenol,2,4-bis(1,1-dimethylethyl), (**C**) Isolongifolene,4,5,9,10-dehydro, (**D**) 9-Methyl-S-octahydroanthracene, (**E**) 9-Methyl-S-octahydrophenanthrene, (**F**) á-Himachalenoxide, (**G**) Longifolenaldehyde, (**H**) ç-Gurjunenepoxide-(1), (**I**) Crocetane, (**J**) Heneicosane, (**K**) 9-n-Hexylheptadecane, (**L**) Octadecane, 3-ethyl-5-(2-ethylbutyl), (**M**) Diisooctyl phthalate, and (**N**) Mono(2-ethylhexyl) phthalate.

**Figure 5:**
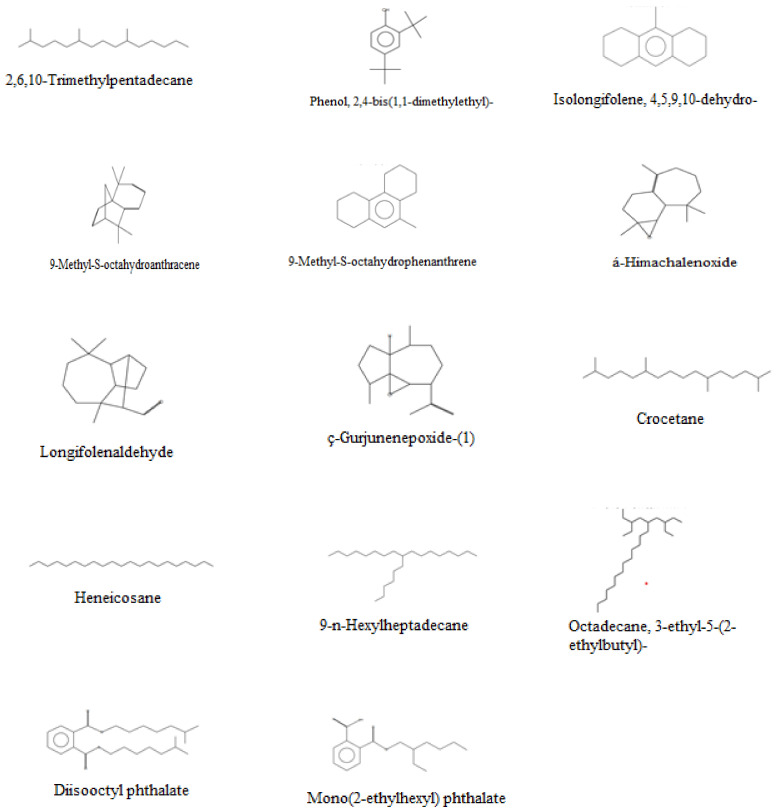
Chemical structures of the major phytochemical compounds identified in *Indigofera linifolia* crude extract.

GC–MS analyses were performed to get insight into the phytoconstituents of a volatile nature. This technique is very rapid and authentic in the determination of volatile compounds in plants; for example, 25 phytochemical substances have been identified in the leaves and rhizomes of *Amomum* *nilgiricum* species in a reported study [22], whereas in the present study we have identified 14 compounds in the selected plant, as given in Table 3. The GC and GC–MS chromatograms are shown in Figure 3 and Figure 4, respectively, whereas their chemical structures are given in Figure 5. The phytochemicals confirmed were: 2,6,10-Trimethylpentadecane, Phenol,2,4-bis(1,1-dimethylethyl), Isolongifolene, 4,5,9,10-dehydro, 9-Methyl-S-octahydroanthracene, 9-Methyl-S-octahydrophenanthrene, á-Himachalenoxide, Longifolenaldehyde, ç-Gurjunenepoxide-(1), Crocetane, Heneicosane, 9-n-Hexylheptadecane, Octadecane, 3-ethyl-5-(2-ethylbutyl), Diisooctyl phthalate, and Mono(2-ethylhexyl) phthalate.

### 2.4. Characterization of the Prepared NPs

#### 2.4.1. FTIR Analysis of the Crude Extract and Fabricated NPs

**Figure 6:**
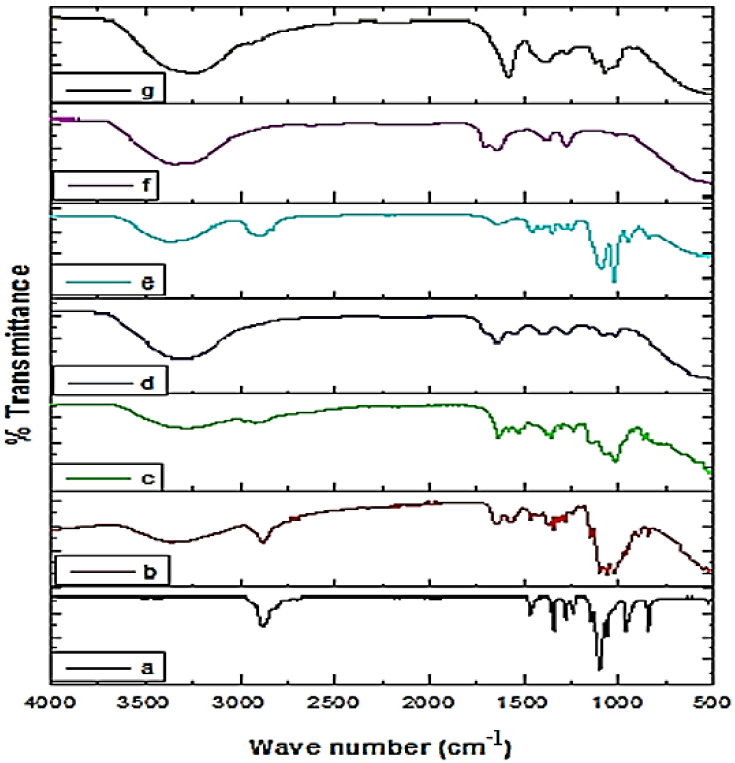
FTIR spectra of PEG (**a**), chitosan (**b**), LGB (**c**), Ext+Ch NPs (**d**), Ext+PEG NPs (**e**), Ext+LBG NPs (**f**), and crude extract (**g**).

FTIR spectroscopy is the most suitable non-destructive spectroscopic technique and has become an attractive method for analyzing pharmaceutical solids [23,24]. The spectra of the prepared NPs and the respective materials are presented in Figure 6. The extract’s spectra showed absorption bands in the range of 3600–2500 cm^−1^, which is characteristic of OH, NH, and carbon–hydrogen bonds and is attributed to the nature of the organic compounds in the extract and the presence of extensive hydrogen bond interactions. The peaks in the region of 1350–1000 cm^−1^ are related to carbon–oxygen bonds (CO) that can be attributed to ether, esters, and carboxylic acids, thus indicating a wide variety of metabolites [23,25]. The peaks that appeared at 1702–1685, 1607–1516, and 788–674 cm^−1^ can be attributed to carbonyl, C = C bonds in the aromatic rings and aromatic CH bonds, respectively [23]. A comparative FTIR spectra study was carried out to investigate the possible interaction of extract-loaded NPs of LGB, PEG, and chitosan. The extract-loaded NPs exhibited peaks shifting, attenuating, and broadening that can be attributed to the establishment of Van der Waal interactions (H-bonding, dipole–dipole interactions, and London dispersion forces) among the raw material mixed [25]. Moreover, the characteristic peaks of individual components in the extract-loaded polymer NPs revealed the successful loading of extract into the fabricated NPs.

#### 2.4.2. TGA of Crude Extract and Prepared NPs

**Figure 7:**
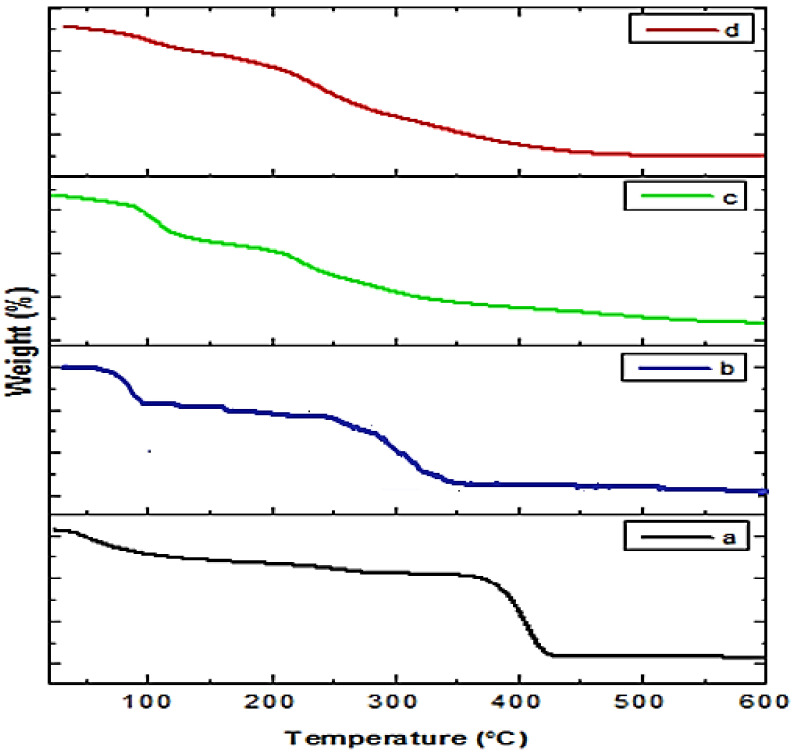
TG thermograms of Ext+Ch NPs (**a**), Ext+PEG NPs (**b**), crude extract (**c**), and Ext+LBG NPs (**d**).

TGA was used to evaluate the thermal decomposition of plant extract-loaded polymer (PEG, LBG, and chitosan) NPs and crude extract. The thermograms of crude extract and extract-loaded NPs are presented in Figure 7. The thermogram of plant extract showed weight loss in different stages. In the first stage, there is a 9% weight loss up to 100 °C that can be attributed to free solvent and volatile products detachment [26]. In the second stage (100–130 °C), a weight loss of 15% observed may be due to the loss of bound solvent molecules and volatile products of the plant extract. In the next two stages (130–200 and 200–600 °C), thermal degradation has occurred, showing the loss of many secondary metabolites, mainly phenolics present in the extract.

*Indigofera linifolia* extract-loaded polymer (PEG, LBG, and chitosan) NPs thermograms showed a different thermal behavior than the plant extract, which may be due to the interaction between plant extract and polymers. The thermograms of Indigofera *linifolia* Ext+LBG and Ext+Ch NPs a showed greater thermal stability than that of the crude extract.

#### 2.4.3. SEM Analysis of Plant Extract-Loaded NPs

**Figure 8:**
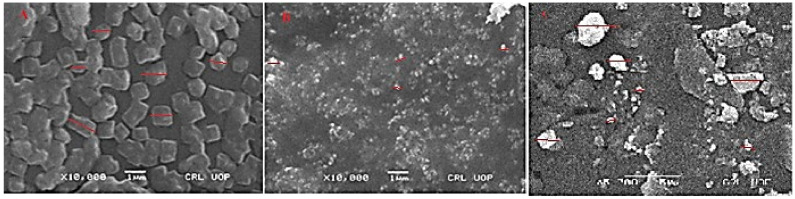
SEM images of Indigofera *linifolia* Ext+PEG NPs (**A**), Ext+Ch NPs (**B**), and Ext+LGB NPs (**C**).

SEM images can directly provide information on the particle size and morphology of NPs. Figure 8 represents the SEM images of the *Indigofera linifolia* leaves extract-loaded polymers (chitosan, PEG, LBG) NPs. The SEM images confirm the formation of plant extract-loaded polymer nanoparticles. The average sizes of the selected particles were in the ranges: Ext+PEG NPs = 450 to 950 nm, Ext+Ch NPs = 250 to 450 nm, and Ext+LBG NPs = 300 to 1500 nm with the morphology of cubical to rectangle shape (Ext+PEG NPs) and spherical to slightly ellipsoid, and irregular shapes (Ext+Ch NPs and Ext+LBG NPs).

### 2.5. Antibacterial Activity of Crude Extract and Prepared NPs

The antibacterial activity of crude methanolic extract, polymers (PEG, chitosan, and LGB), and the synthesized NPs are shown in Table 4. All tested samples showed activity against the tested bacterial strains (*Escherichia coli*, *Klebsiella pneumonia*, and *Salmonella typhi*). The maximum zone of inhibition was detected for Ext+PEG NPs against the tested bacterial strains, which were 17 ± 0.3 mm, 16.6 ± 0.6 mm, and 14 ± 04 mm, respectively. The methanolic leaves extract demonstrated a good antibacterial activity, determined through the cup–plate agar diffusion method, showing that methanol is an effective organic solvent for extracting bioactive plant components. It has been reported that in a related plant, *Indigofera tinctoria* methanol extract has antibacterial activity against *Staphylococcus aureus*, *Streptococcus pyogenes*, and *Bacillus pumilus* [27]. In another study, methanol extract of the same plant leaves have exhibited antibacterial action against methicillin-resistant *Staphylococcus aureus*, *Escherichia* *faecalis*, *Moraxella catarrhalis*, *Haemophilus influenzae*, and anaerobes, as pointed out by Vijayan et al. [28]. *Tradescantia zebrina* methanol leaf extract had the highest antioxidant content and activity, with an antibacterial activity against six Gram-positive and two Gram-negative bacteria in concentrations of 5–10 mg/mL [29]. NPs systems are colloidal particles that range in size from 1 to 1000 nm in diameter [30]. They are tiny enough to penetrate through biological barriers, internalize target cells, and influence a variety of cellular processes due to their high surface/volume ratio and association with structural sizes of biological components [31]. It has been reported that extract-loaded NPs can protect cargo from biodegradation, preserving bioactivity, increase circulation durations, enable regulated release, and maintain efficacy at the target site after utilizing in fewer dosages rather than if utilized in the free form [32].

### 2.6. Determination of Free Radical Scavenging Activity of Crude Extract and Prepared NPs by DPPH Assay

The antioxidant potential of extract, Ext+Ch NPs, Ext+PEG NPs, and Ext+LGB NPs of *Indigofera linifolia* leaves was studied by 2,2-Diphenyle-1 picrylhydrazyl (DPPH) at different concentrations ranging from 1000, 500, 250, 125, to 62.5 μg/mL. The antioxidant activity is based on the ability of the electron donor to scavenge DPPH, a stable free radical upon which the color changes from purple to yellow. The percent free radical scavenging potential was 87.83 ± 0.66, 76.32 ± 0.24, 88.81 ± 0.39, 84.59 ± 0.36, and 94.88 ± 0.56 at the highest concentration for crude extract, Ext+Ch, Ext+PEG, and Ext+LGB, respectively, with corresponding IC_50_ values of 53, 45, 41, and 39 μg/mL. Ascorbic acid was used as a standard and its IC_50_ value was 30 μg/mL. The potent DPPH scavenging activity (Table 5) of the methanolic leaf extracts and NPs of *Indigofera linifolia* may be attributable to the neutralization of free radicals by the transfer of hydrogen or an electron [33]. It is an established fact that in the human body the mechanism behind the inhibition of lipid oxidation is free radical scavenging [34]. Srinivasan et al. [35] and Muhammad et al. [36] have found that methanolic leaf and root extracts of the same genus plants have the highest free radical scavenging activity. Phytochemicals such as phenolics have been found in abundance in plants, and different health benefits such as antioxidant activity have been claimed by virtue of such rich phytochemical compositions [37,38]. Mahmood et al. [39] also found that the antioxidant properties of natural resources were highly associated with their primary chemical content, such as polyphenols. Curcumin encapsulated by nano and pickering emulsion stabilized by chitosan-tripolyphosphate nanoparticles have been evaluated for their radical scavenging activity and higher results were recorded for the formulations as compared to free curcumin, which dictates the protective effect of the emulsion systems on the antioxidant activity of curcumin bioactive compounds [40].

### 2.7. In Vitro Antidiabetic Activities of Crude Extract and Prepared NPs

Diabetes mellitus, a metabolic disorder with multiple etiologies, can be characterized by a failure of glucose homeostasis as well as disturbances in carbohydrate, fat, and protein metabolism as a result of defects in insulin secretion and/or insulin action [41], and is one of the world’s major public health problems. According to research by the International Diabetes Federation, the raised blood glucose level in humans is the third most important risk factor of premature death worldwide, after high blood pressure and cigarette use [42]. Previously, it has been reported that *Ajuga remota* leaf extracts have the capability to lower high fasting blood glucose levels [43]. The presence of well-known antioxidant phytochemicals such as flavonoids, polyphenols, and tannins, which serve as free radical scavengers, may explain the antidiabetic benefits of *Ajuga remota* leaf extracts [44,45]. These antioxidants were thought to have an insulin-mimetic effect on peripheral tissues, either by stimulating the regeneration process or by regulating pancreatic insulin secretion from existing cells. Other processes may also be involved in addition to this one and may have a significant role in the decreasing of blood glucose levels that needs to be explored. Increasing the rate of glucose release from the circulation by accelerating filtration and renal excretion, as well as increasing glucose release through enhanced metabolism or integration into fat deposits, a process involving the pancreas to produce insulin, are just a few of the other options [44]. Based on previous literature, we have evaluated the antidiabetic activity of leaves crude extract and prepared NPs in this as described below.

#### 2.7.1. In Vitro α-Glucosidase Enzyme Inhibition

The percent inhibitory activities along with IC_50_ values of the crude, Ext+Ch NPs, Ext+LGB NPs, and Ext+LGB NPs calculated against the α-glucosidase enzyme are given in Table 6. The % α-glucosidase inhibition of crude and Ext+Ch NPs, Ext+PEG NPs, and Ext+LGB NPs were 66.15 ± 0.92, 72.58 ± 0.84, 63.15 ± 0.45, 77.59 ± 0.20 µg/mL, respectively, at their highest concentration (1000 µg/mL) as compared to that of acarbose, for which the recorded activity at the mentioned highest concentration was 92.11 ± 0.84 µg/mL. The highest inhibition of the enzymes’ activity was observed for Ext+LGB NPs.

#### 2.7.2. In Vitro α-Amylase Enzyme Inhibition

The IC_50_ values along with percent inhibition of the crude and Ext+Ch NPs, Ext+PEG NPs, and Ext+LGB NPs against the α-amylase enzyme are also given in Table 6. The percent inhibition of the activity of the enzyme of crude, Ext+Ch NPs, Ext+PEG NPs, and Ext+LGB NPs at their highest concentration recorded was: 71.16 ± 0.13, 79.72 ± 0.89, 67.97 ± 0.09, 83.37 ± 0.21 µg/mL respectively, in comparison to that of acarbose (86.72 ± 1.14 µg/mL). The maximum inhibition of this enzyme was observed for Ext+LGB NPs.

## 3. Materials and Methods

### 3.1. Chemicals Used

The standard antioxidant quercetin was obtained from Sigma-Aldrich, France, while Folin–Ciocalteu (F-C) reagent and sodium carbonate, 2,2-Diphenyle-1 picrylhydrazyl (DPPH) were from Sigma-Aldrich CHEMIE GmbH, USA, whereas aluminum chloride, sodium nitrite, sodium hydroxide, ethanol, methanol, and ascorbic acid were purchased from Sigma-Aldrich, Germany. Table 7 shows some basic information about the polymer used. All the chemicals used were of analytical grade except the HPLC-grade solvents used in HPLC analysis that were purchased from Sigma-Aldrich, Germany. They were used as such without any further purification.

### 3.2. Plant Sample Collection

The leaves of the *Indigofera linifolia* were collected from Jabban Mountain, Malakand Khyber Pakhtunkhwa, Pakistan, in its harvesting season, in the month of October 2020. After collection, the leaves were cleaned with tap water and then washed with distilled water.

### 3.3. Identification

The taxonomist in the Department of Botany, University of Malakand, identified the plant and a specimen was kept in the herbarium sheet of plants with a voucher specimen; BGUOM; 132, deposited in the herbarium of Malakand University.

### 3.4. Crude Extract Preparation

Fresh leaves of *Indigofera linifolia* were thoroughly washed to remove dust, air dried at room temperature for a month, and crushed into fine powder. The powdered sample (200 g) was soaked in methanol with continuous vigorous shaking for six days. The mixture was filtered through the Whatman filter paper, and the filtrate was again soaked in methanol for additional six days. The filtrates from both the steps were combined and concentrated in a rotary evaporator (Rotavapor R-200 Buchi, Flawil, Switzerland) at low pressure and 40 °C temperature.

### 3.5. Preliminary Phytochemical Analysis of a Crude Extract of Indigofera Linifolia

To identify major phytochemical groups in the crude extract of selected plant-specific tests for alkaloids, flavonoids [46], glycosides [47] tannins, tannins [48], and terpenoids [49] were carried out following previously reported assays.

### 3.6. Determination of Total Phenolic Content (TPC) and Total Flavonoid Contents (TFC)

The TPC in *Indigofera linifolia* crude extract was determined using the F-C reagent as per described procedure [46] while the TFC in the sample was estimated as milligrams of quercetin mg QE/g of the dry sample [47] following reported assays in the literature. The experiments were performed in triplicate and average values were reported.

### 3.7. HPLC-UV Analysis of the Crude Extract

For HPLC analysis, 1 g methanolic extract was added into a mixture of water and methanol in 1:1 ratio, following standard protocol [50]. The mixture was heated at 50 °C for 1 h. The mixture was then filtrated using Sterile Syringe Filter 0.45 µm and poured into HPLC vials. For phytochemicals identification, the sample was loaded on HPLC Agilent 1260 system, equipped with basic parts such as an autosampler, quaternary pump, degasser, and ultraviolet detector. The C18 column was used for the separation of phytoconstituents while the detector was operated at 320 nm. Retention times of unknown samples and available standards were used for the identification of compounds.

### 3.8. GC–MS Analysis

According to the previously reported protocol, the volatile phytoconstituents in a crude extract of *Indigofera linifolia* were analyzed through GC–MS; Agilent technology USA [51]. The system was equipped with an FID detector for the identification of phytochemicals. Compounds were identified by comparing their mass spectra and retention times with pure compounds (standards) available at Wiley and NIST libraries.

### 3.9. Locust Bean Gum (LBG) Extraction

The carob seeds’ lengths between 5.5 and 6 mm and thickness between 3.5 and 4 mm were collected from the garden of the University of Malakand, Pakistan. As a pre-treatment, whole carob seeds (50 g) were immersed in 600 mL of boiling water at 100 °C for 1 h. During this pre-treatment, the seeds swelled without tegumental disruption. Seeds were removed from the water, washed, broken, and separated manually from the endosperm. The germ from endosperms was dried at 100 °C for 1–2 h to obtain a constant weight. The endosperms were then crushed and sifted with a 0.125 mm sieve to obtain LBG flour [52].

### 3.10. Synthesis of NPs

Indigofera linifolia leaf extract NPs were prepared by using three polymers; chitosan, PEG, and LGB. Chitosan solution was made by gently adding 40 mg of chitosan (deacetylation degree 85%, M) to 1% (*v*/*v*) acetic acid. To obtain a homogenous solution, the solution was constantly agitated at 7000 rpm using a homogenizer (BagMixer 400 W, Shanghai, China). To obtain nanoparticles, about 10% of plant aqueous extract relative to the chitosan content was added. An ionic crosslinking agent, sodium tripolyphosphate (TPP), was employed to crosslink chitosan as nanoparticles. In this regard, 8 mL of 0.1% TPP solution produced in distilled water was added dropwise to the chitosan solution while homogenizing at a continuous speed of 7000 rpm at room temperature for 2 h. Finally, the extract-loaded NPs were centrifuged (SelectSpin Spectra 6C) at 15,000 rpm for 30 min to remove them from the solution and freeze-dried to get nanoparticles in powder form. The LGB (2 mg mL^−^^1^) solution was prepared in deionized water and mixed for 5 min at 800 rpm. Extract (5 mg mL^−^^1^) was dissolved in methanol at optimal concentrations (antisolvent). Using a syringe pump device (ISPLab01), extract (0.5 mL min^−1^) was introduced to the LGB solution. The solution was agitated at 800 rpm for 10 min after the organic phase was added. Using a sonicator (Elmasonic E30H, Singen, Germany) the solution was sonicated for five minutes. The leftover nanoparticle suspensions were centrifuged at 9000 rpm for 30 min, with the supernatant being discarded. Approximately 10 mg mL^−^^1^ PEG (6000) was dissolved in deionized water. Extract (5 mg mL^−1^) was dissolved in methanol to make the solvent solution. The methanolic extract solution was added dropwise to antisolvent and then sonicated for 5 min. The solvent was then evaporated under vacuum at 45 °C using rotary evaporator.

#### Characterization of NPs

Standard characterization techniques *viz*., Fourier transform infrared (FT-IR) spectroscopy, scanning electron microscopic (SEM), and thermogravimetric (TGA) analyses were performed to characterize the formulated nanoparticles. FTIR analyses were performed using FTIR spectrophotometer (PerkinElmer spectrum-10.5.1). The FTIR spectra of crude extract and prepared NPs were recorded in the range of 4000–400 cm^−1^. Morphology and size of NPs particles were determined using scanning electron microscopic (SEM) analyses. Samples of nanoparticles were mounted on metal stubs, gold-coated under vacuum, and then examined on a JEOL-5600 Lv microscope (Joel, Tokyo, Japan). Thermal gravimetric studies of crude extract and prepared NPs were performed with the help of Diamond Series TG/DTA Perkin Elmer, USA analyzer using Al_2_O_3_ as reference. The sample was heated at a rate of 10 °C/min in the range of 40–300 °C.

### 3.11. Antimicrobial Activity of Extract and Prepared NPs

The antibacterial activity of extract, polymers, and NPs were screened out using the agar well diffusion method [53]. In the center of a sterile Petri dish, one ml of new bacterial culture was pipetted. Following solidification, wells were drilled into agar plates containing inoculums with a sterile cork borer (5 mm in diameter). After that, 100 μL of each extract (20% *w*/*v*) was applied to the appropriate wells. The plates were chilled for 30 min to allow the extracts to fully diffuse into the agar. The plates were then incubated for 18 h at 37 °C. After the incubation time, the zone of inhibition (including the well width) was measured to determine antimicrobial activity. Against each strain the experiments have been performed in three replicates and average zone of inhibition were recorded.

### 3.12. Determination of Free Radical Scavenging Activity by DPPH Assay

Total free radical scavenging ability of leaf extract and NPs were measured using the previously reported method [54] with minor modifications utilizing the stable DPPH radical, which has an absorption maximum at 517 nm. A solution of 0.1 mM DPPH in methanol was prepared and 2.4 mL of this solution was mixed with 1.6 mL of extract and prepared NPs in methanol at different concentrations (1000, 500, 250, 125, and 62.5 μg mL^−1^). The mixture was vigorously mixed and stored at room temperature in the dark for 30 min. The reaction mixture absorbance was measured spectrophotometrically at 517 nm. The absorbance of the DPPH radical in the absence of antioxidant, or blank, was also tested. All of the tests were carried out in triplicate. The following equation [4] was used to calculate the ability to scavenge the DPPH radical.

(1) $$\mathrm{DPPH}\;\mathrm{Scavenged}\;\left(\%\right)=\frac{AB-AA}{AB}\times 100$$

where, *AB* is the blank sample absorbance at t = 0 min and *AA* is the absorbance of antioxidant containing sample at t = 30 min. The IC_50_ values were estimated from a plot of percent DPPH verses sample concentrations. The same procedure was used for standard antioxidant (ascorbic acid). The experiments were performed in three replicates.

### 3.13. Antidiabetic Potential of Extract and NPs

The antidiabetic potential of extract and NPs were determined through monitoring their inhibitory potentials against two important carbohydrate metabolizing enzymes; alpha-amylase and alpha-glucosidase. The extract and NPs were tested for alpha-amylase inhibitory activity using the reported method with minimal modifications [55]. A reaction mixture containing 50 μL phosphate buffer (100 mM, pH = 6.8), 10 μL alpha–amylase (2 U/mL), and 20 μL extract and NPs (1000, 500, 250, 125 and 62.5 μg mL−1) was incubated at 37 °C for 20 min in a 96-well plate. Then, as a substrate, 20 μL of 1 percent soluble starch (100 mM phosphate buffer pH 6.8) was added and incubated for another 30 min at 37 °C; 100 μL of dinitro salicylic acid coloring reagent was added and boiled for 10 min. Using a multiplate reader, the absorbance of the resultant combination was measured at 540 nm (Multiska thermo scientific, version 1.00.40, Thermo Fisher Scientific, Waltham, MA, USA). Acarbose was employed as a standard at varied concentrations (1000–62.5 μg/mL). The substance that was not tested (extract and fractions) was set up in parallel as a control, and each experiment was done in triplicates. The results were calculated using the formula, which were expressed as a percentage inhibition.

(2) $$\mathrm{Inhibition}\;\left(\%\right)=\left(1-\frac{As}{Ac}\right)\times 100$$

where, *As* is the absorbance in the presence of test substance and *Ac* is the absorbance of control.

The inhibitory activity of alpha-glucosidase by extract and NPs were determined using the reported method with slight modifications [55]. A reaction mixture containing 50 μL phosphate buffer (100 mM, pH = 6.8), 10 μL alpha-glucosidase (1 U/mL), and 20 μL of different concentrations of extract and NPs (1000, 500, 250, 125, and 62.5 μg/mL) were incubated at 37 °C for 15 min in a 96-well plate. Then, as a substrate, 20 μL 4-Nitrophenyl-β-D-glucopyranoside (5 mM) was added to each sample and incubated for another 20 min at 37 °C. The process was stopped by the addition of 50 μL of Na2 CO3 (0.1 M). Using a multiplate reader, the absorbance of the emitted p-nitrophenol was measured at 405 nm. As a control, acarbose was used at varying concentrations (1000–62.5 μg mL^−1^). A control experiment with no test drug was set up in parallel, and each experiment was carried out in triplicate. The percent inhibitions were calculated using Equation (2). The experiments were performed in triplicate and average values were reported.

## 4. Conclusions

*Indigofera linifolia* leaf crude methanolic extract was used as a raw material in the preparation of nanoparticles in this study. The crude extract of the leaf extract was encapsulated in three different polymers, viz., chitosan, locust bean gum, and polyethylene glycol to improve their biological potency and efficacy. The leaf extract-loaded polymer, i.e., Ext+Ch, Ext+LBG, and Ext+PEG, were converted into NPs following standard procedures. The crude extract and the prepared NPs were then assessed for their potential biological activities such as antibacterial, antioxidant, and antidiabetic. The highest antibacterial activity was observed for Ext+PEG NPs, whereas a higher antioxidant activity was recorded for Ext+LBG NPs assessed through the DPPH assay. The antidiabetic activity was determined against α-glucosidase and α-amylase. Ext+LGB inhibited both enzymes’ activity with IC_50_ values of 83 and 78 µg/mL, respectively, which was the most potent among the tested samples. The performed biological activities of the formulations were compared with that of extract and in each case, the formulations produced pronounced effects rather than the extract. The prepared NPs may thus be used or considered to be used as a remedy of microbial infections, oxidative stress, and diabetes mellites complications. The findings of this study in terms of observed biological activities in the prepared formulations could be looked at in the perspective of new drug discovery.

## Figures and Tables

**Table 1:** Preliminary qualitative phytochemical screening of *Indigofera linifolia* crude extract.

Phytochemical	Reagent	Observation	Results
Flavonoids	Ferric chloride	Appearance of yellow color and after the addition of HCl become colorless.	+
Glycosides	Keller Killiani	Formation of a red to brown layer.	+
Tannins	Gelatin	Brownish-green precipitates.	+
Triterpenoids	Liebermann Burchard	Reddish-brown boundary.	+

**Table 2:** Identified phytochemicals in the crude extract of *Indigofera linifolia* (identified from HPLC-UV chromatogram).

Retention Time (min)	Phytochemical Compounds	Sample Peak Area	Identification Reference
10.329	Mandelic acid	55.548	Ref. Stand
11.273	Bis-HHDP-hex(pedunculagin)	797.264	[19]
12.478	Caffeic acid	510.522	Ref. Stand
16.437	Hydroxy bezoic acid	38.4805	Ref. Stand
20.978	Chlorogenic acid	91.0721	Ref. Stand
22.672	Morin	32.6104	Ref. Stand
23.464	Syringic acid	100.907	[20]
25.087	5-0-dicaffeoylquinic acid	42.0698	[19]
25.545	Kaempferol-3-(caffeoyl-diglucoside)-7-rhamnosyl	50.79067	[19]
27.822	Kaempferol-3-(p-coumaroyl-diglucoside)-7-glucoside	15.8819	[19]
30.089	Quercetin	131.189	Ref. Stand
31.027	Qurcetin-3-(caffeoyldiglucoside)-7-glucoside	24.0199	[19]
34.843	Quercitin-3-O-rutinoside	47.3937	[19]
37.845	Glucose	4.6713	[21]

**Table 3:** Major phytochemical compounds identified in *Indigofera linifolia* and their various parameters.

Retention Time (min)	Molecular Mass (g/mol)	Molecular Formula	%Area	Area	Peak Height	Compound Name
13.08	254	C_18_H_38_	0.00	258,385.77	93,693.15	2,6,10-Trimethylpentadecane
13.93	206	C_14_H_22_O	0.08	5,736,551.10	1,410,675.04	Phenol, 2,4-bis(1,1-dimethylethyl)-
14.30	200	C_15_H_20_	0.01	466,139.33	131,832.12	Isolongifolene, 4,5,9,10-dehydro-
14.30	200	C_15_H_20_	0.01	466,139.33	131,832.12	9-Methyl-S-octahydroanthracene
14.30	200	C_15_H_20_	0.01	466,139.33	131,832.12	9-Methyl-S-octahydrophenanthrene
15.95	220	C_15_H_24_O	0.01	363,808.41	123,197.77	á-Himachalenoxide
15.95	220	C_15_H_24_O	0.01	363,808.41	123,197.77	Longifolenaldehyde
15.95	220	C_15_H_24_O	0.01	363,808.41	123197.77	ç-Gurjunenepoxide-(1)
17.23	282	C_20_H_42_	0.02	1,260,254.99	450,404.86	Crocetane
17.23	296	C_20_H_42_	0.02	1,260,254.99	450,404.86	Heneicosane
18.68	324	C_23_H_48_	0.01	371,092.40	137,687.26	9-n-Hexylheptadecane
20.31	366	C_26_H_54_	0.00	145,679.34	86,758.74	Octadecane, 3-ethyl-5-(2-ethylbutyl)-
23.20	390	C_24_H_38_O_4_	0.26	18,393,028.17	7,282,283.86	Diisooctyl phthalate
23.20	278	C_16_H_22_O_4_	0.26	18,393,028.17	7,282,283.86	Mono(2-ethylhexyl) phthalate

**Table 4:** Antibacterial potential of crude extract of *Indigofera linifolia*, polymers (PEG, chitosan, and LGB), Ext+Ch NPs, Ext+ PEG NPs, and Ext+LGB NPs.

Microbial Strain				Zone of Inhibition (mm)			
	Crude Extract	PEG	Chitosan	LGB	Ext+Ch NPs	Ext+PEG NPs	Ext+LGB NPs
*Escherichia coli*	13.2 ± 0.9	8.0 ± 0.5	8.3 ± 0.6	8.0 ± 0.5	12.6 ± 0.4	17 ± 0.3	10 ± 0.5
*Klebsiella pneumoniae*	10.8 ± 0.4	9.1 ± 0.7	8.2 ± 0.5	7.8 ± 0.7	12.6 ± 0.6	16.6 ± 0.6	11 ± 0.3
*Salmonella typhi*	8.4 ± 0.2	7.9 ± 0.4	9.6 ± 0.7	7.4 ± 0.4	11 ± 0.7	14 ± 0.4	9 ± 0.5

**Table 5:** Antioxidant potential of *Indigofera linifolia* crude extracts and prepared nanoparticles.

S. No	Sample	Concentration (µg/mL)	DPPH% Scavenging	IC_50_
1	Crude extract	1000	87.83 ± 0.66	53
		500	83.27 ± 0.84	
		250	74.19 ± 0.24	
		125	60.49 ± 0.58	
		62.5	57.16 ± 0.49	
2	Ext+Ch NPs	1000	76.32 ± 0.24	45
		500	68.64 ± 0.41	
		250	64.27 ± 0.58	
		125	57.47 ± 0.89	
		62.5	53.52 ± 0.82	
3	Ext+PEG NPs	1000	88.81 ± 0.39	41
		500	81.36 ± 0.45	
		250	75.72 ± 0.34	
		125	71.47 ± 0.31	
		62.5	63.71 ± 0.23	
4	Ext+LGB NPs	1000	84.59 ± 0.36	39
		500	81.81 ± 0.39	
		250	73.84 ± 0.58	
		125	68.73 ± 0.91	
		62.5	62.38 ± 0.89	
5	Ascorbic acid	1000	94.88 ± 0.56	30
		500	86.59 ± 0.45	
		250	78.64 ± 0.76	
		125	69.14 ± 0.25	
		62.5	62.87 ± 0.53	

**Table 6:** α-glucosidase and α-amylase enzyme inhibition of crude and Ext+Ch NPs, Ext+PEG NPs, and Ext+LGB NPs at the highest concentrations.

Sample	Concentration (µg/mL)	% α-Glucosidase Inhibition Mean ± SEM	IC_50_ (µg/mL)	% α-Amylase Inhibition	IC_50_ (µg/mL)
				Mean ± SEM	
Crude extract	1000	66.15 ± 0.92	120	71.16 ± 0.13	104
	500	60.74 ± 0.45		64.41 ± 0.71	
	250	58.99 ± 0.18		60.06 ± 0.23	
	125	51.31 ± 0.15		54.72 ± 0.91	
	62.5	43.37 ± 1.02		49.67 ± 0.37	
Ext+Ch NPs	1000	72.58 ± 0.84	100	79.72 ± 0.89	86
	500	69.07 ± 0.98		73.22 ± 0.16	
	250	67.98 ± 0.64		67.02 ± 0.23	
	125	51.75 ± 0.56		61.97 ± 0.56	
	62.5	49.78 ± 1.78		54.58 ± 0.78	
Ext+PEG	1000	63.15 ± 0.45	126	67.97 ± 0.09	112
	500	57.45 ± 0.73		61.27 ± 0.30	
	250	55.04 ± 0.03		56.22 ± 0.13	
	125	39.69 ± 1.03		51.82 ± 0.07	
	62.5	37.06 ± 1.08		47.18 ± 0.15	
Ext+LGB	1000	77.59 ± 0.20	83	83.37 ± 0.21	78
	500	71.62 ± 0.09		76.29 ± 0.08	
	250	64.60 ± 0.65		71.57 ± 0.34	
	125	58.46 ± 0.34		68.47 ± 0.36	
	62.5	54.92 ± 1.34		61.88 ± 0.67	
Acarbose	1000	92.11 ± 0.84	69	86.72 ± 1.14	74
	500	86.13 ± 1.34		80.54 ± 0.54	
	250	80.47± 1.82		73.76 ± 1.02	
	125	76.32 ± 1.22		69.22 ± 2.02	
	62.5	71.18 ± 2.02		64.28 ± 1.75	

**Table 7:** Basic information about the polymers used.

Name of Polymer	Formula	Molecular Weight	Supplier
PEG 6000	H(OCH_2_CH_2_)_n_OH	5000–7500	Sigma-Aldrich, Germany
Chitosan	C_56_H_103_N9O_39_	1526	Sigma-Aldrich, Germany
LBG (extract from seed)	--	--	--

## Sample Availability Statement

Samples of the compounds are not available from the authors.
